# Genetic Diversity of *Mycobacterium tuberculosis* Complex Isolated from Tuberculosis Patients in Bahir Dar City and Its Surroundings, Northwest Ethiopia

**DOI:** 10.1155/2015/174732

**Published:** 2015-09-28

**Authors:** Anwar Nuru, Gezahegne Mamo, Adane Worku, Aschalew Admasu, Girmay Medhin, Rembert Pieper, Gobena Ameni

**Affiliations:** ^1^Aklilu Lemma Institute of Pathobiology, Addis Ababa University, P.O. Box 1176, Addis Ababa, Ethiopia; ^2^Faculty of Veterinary Medicine, University of Gondar, P.O. Box 346, Gondar, Ethiopia; ^3^College of Veterinary Medicine and Agriculture, Addis Ababa University, P.O. Box 34, Debre Zeit, Ethiopia; ^4^Bahir Dar Regional Health Research Laboratory Centre, P.O. Box 641, Bahir Dar, Ethiopia; ^5^J. Craig Venter Institute, 9704 Medical Center Drive, Rockville, MD, USA

## Abstract

The knowledge of the diversity of strains of *Mycobacterium tuberculosis* complex (MTBC) species in a specific geographical region can contribute to the control of tuberculosis (TB). This study was conducted to identify the MTBC isolates to the species and spoligotype international type (SIT) level by spoligotyping. A total of 168 MTBC isolates were recovered from TB patients, spoligotyped, and their patterns were compared with those of the strains registered in the SITVIT2 database. Of 168 isolates spoligotyped, 89 patterns were identified. Ninety-eight isolates were clustered into 19 strain groups with clustering percentage of 58.3%. Forty-four strains matched the preexisting SITs in the SITVIT2 database. The dominant strains were SIT289, SIT134, and SIT3411, comprising 16.7% (28/168), 7.14% (12/168), and 4.76% (8/168) of the isolates, respectively. Euro-American (51.2%), East-African-Indian (34.5%), and *M. africanum* (9.52%) were the major lineages identified. Two strains of *M. bovis* were isolated from TB lymphadenitis cases. The high percentage of clustered strains of *M. tuberculosis* could suggest that a small number of lineages of *M. tuberculosis* are causing the disease in the area while isolation of *M. bovis* could suggest its zoonotic potential. Additionally, identification of *M. africanum* requires further confirmation by tools with a better discriminatory power.

## 1. Introduction

The incidence of TB has continued to increase in many parts of the world [1]. An estimated 9 million people developed TB in 2013, which is 6% greater than that reported in 2012. A quarter of these cases occurred in the African region [1]. About 1.5 million deaths were attributed to TB globally, of which approximately 75% occurred in Africa and Southeast Asia [1]. The 22 high TB burden countries (HBCs) collectively accounted for 82% of all the estimated TB incidences worldwide [1]. Ethiopia is among the top ten HBCs with an estimated incidence rate of 224 patients per 100,000 population [1], which is much greater than 126/100,000 reported globally in 2013 [1]. Moreover, TB is the second most common cause of hospital deaths in the country [2]. TB lymphadenitis in cervical lymph nodes is also common in Ethiopia and accounted for 33% of all new TB cases [3], which is greater than the global average of 15% [4]. According to the 2013 Heal TB Project Report of 2014, the incidence of TB in the Amhara Region (present study area) for one year (October 2012 to September 2013) was estimated to be 247 per 100,000 population (unpublished, URL: http://pdf.usaid.gov/pdf%20docs/pa00jn8p.pdf), which is higher than the national incidence rate of Ethiopia during the same year. However, little or no information is available on the type of MTBC species and strains causing the disease in the study area.

Thus, identification of strains circulating in a certain geographic region using molecular tools can contribute to the TB control program of that region. Various molecular epidemiology methods allow identifying mycobacterial strains and tracking the transmission of TB in different geographical regions [5]. For example, spoligotyping is widely used for identification of* M. tuberculosis* [6]. Hence, the purpose of this study was to identify the MTBC isolates to the species and SIT level by the use of spoligotyping.

## 2. Materials and Methods

### 2.1. Study Area

The study was conducted in Bahir Dar city and its surrounding districts. Bahir Dar is a city in the highland in northwest of Ethiopia and is the capital city of the Amhara Regional Administrative State. The city is located at geographical coordinates of 11°38′ north in latitude and 37°15′ east in longitude. It has an elevation of 1830 meters above sea level and is characterized by hot and humid weather with an average temperature of 29°C. The population size of the city and its surrounding is 221,991, of which 180,174 (81.2%) are residing in the Bahir Dar city [7]. To seek a better economic situation, rural inhabitants, particularly those living in a radius of 60 km around the city, recently migrated and caused the rapid rise in population in Bahir Dar city [8]. The migration led to homelessness and poverty, as the anticipated job opportunities were not realized. In addition, crowded living conditions, lack of ventilation in temporary housing, malnutrition, and lack of education facilitated the spread of TB [9].

### 2.2. Study Subjects

Smear positive pulmonary TB (PTB) and extrapulmonary TB (EPTB) patients, who were diagnosed as TB cases between September 2012 and January 2014 at Felegehiwot Referral and GAMBY General Hospitals, were included in this study. High TB patient flow, existence of better diagnostic facilities, and skilled human resource were the major reasons for selecting the specified health facilities. The average TB case flow in these two study hospitals over four years (2010–2013) was 321 as assessed from the respective hospital records. TB patients who visited these health facilities during the study period were enrolled in the study, excluding those below 18 years of age and those who had started treatment prior to launching the study. Children under the age of 18 years were not included as (1) the study was not a pure epidemiological study and its main objective was the identification of strains circulating in the study area, (2) it was not easy to obtain consent from a family member or a guardian for children below 18 years of age, (3) collection of sputum samples can be difficult in children, and (4) systematic differences of MTBC strains comparing the adult population with children were not expected.

### 2.3. Sample and Data Collection

A structured questionnaire was used to collect data from all study subjects. These data included patient origin, age, sex, household size, TB category, clinical presentation, and family history of TB infections. Clinical examination of patients suspected to be infected with TB was performed by the attending physicians. Sputum samples submitted for the routine Ziehl Neelsen staining for diagnostic purpose were used for bacteriological examination. Similarly, fine needle aspirates (FNA) collected by a pathologist for the routine diagnosis of TB were used for mycobacterial culture.

### 2.4. Mycobacterium Culture

Isolation of mycobacteria was made on Lowenstein-Jensen (LJ) medium using the procedure described by the National TB and Leprosy Control Programme Guideline [10] that was adopted from WHO guideline [11]. Both sputum and FNA samples were cultured at the Bahir Dar Regional Health Research Laboratory Centre. Briefly, sputum or FNA samples were homogenized and decontaminated with an equal volume of 4% NaOH by centrifugation at 3000 rpm for 15 minutes at room temperature. The supernatant was decanted while the sediment was neutralized with 2 N HCl using phenol red as an indicator. Neutralization was achieved when the color of the solution changed from purple to yellow. About 100 *µ*L of the suspension was inoculated on four sterile LJ medium slopes (two were supplemented with pyruvate and the other two with glycerol) and then incubated at 37°C with weekly examination for growth. Specimens without colonies at eighth week after culturing were considered as negative. Specimens with growth of colonies were examined by Ziehl Neelsen microscopy. AFB positive colonies were harvested and resuspended in 200 *µ*L sterile distilled water. Thereafter, they were inactivated by heating at 80°C for 45 minutes in a water bath and transported to Aklilu Lemma Institute of Pathobiology (ALIPB), Addis Ababa University, for spoligotyping.

### 2.5. Spoligotyping

All of the 168 isolates were characterized by spoligotyping as previously described by Kamerbeek et al. [12] following the instruction supplied with the spoligotyping kit (Ocimum Biosolutions Company, Ijsselstein, The Netherlands). Briefly, the direct repeat (DR) region of the isolate was amplified using DRa and DRb primers. The amplified biotinylated products were hybridized with a set of 43 oligonucleotides covalently bound to a membrane (Animal and Plant Health Agency, Great Britain). Known strains of* M. bovis* and* M. tuberculosis* H37Rv were used as positive controls, whereas Qiagen water (Qiagen Company, Germany) was used as a negative control. Hybridized DNA was detected by the enhanced chemiluminescence method. Images were captured by exposure to X-ray film (Hyperfilm ECL, Amersham) as specified by the manufacturer's instruction. The presence and absence of spacers were visualized on film as black and white squares, respectively.

### 2.6. Comparison of Experimental Data with the SITVIT2 Database

The spoligotype patterns were converted into binary and octal formats and entered into the open source spoligotype database available at the website http://www.pasteur-guadeloupe.fr:8081/SITVIT_ONLINE/tools.jsp. The shared international spoligotype (SIT) number and lineages/sublineages were retrieved from the database. The results were compared with the existing designations in the SITVIT2 database of Institute Pasteur de la Guadeloupe. Two or more mycobacterial isolates sharing a spoligotype pattern in the study were identified as a cluster, whereas single spoligotype patterns in the study were recognized as unique. Strains matching a preexisting pattern in the SITVIT2 database were identified with the SIT number, whereas strains for which SIT numbers were not found from the database were considered as orphan strains. In addition, the online tool “Run TB-Lineage” (http://tbinsight.cs.rpi.edu/run_tb_lineage.html) was also used to predict the major lineages to which the strains belong by a conformal Bayesian network (CBN) analysis.

### 2.7. Statistical Analysis

The statistical analysis was performed using STATA software version 12 [13]. Descriptive statistics were used to depict the demographic and clinical variables. Chi-square or Fisher's exact tests were used to evaluate the association of clusters and major lineages with selected patient characteristics. *P* values of less than 0.05 were considered statistically significant.

### 2.8. Ethical Considerations

Ethical clearance was obtained from Ethical Review Board (Ref. number IRB/05-02/2013) of the Aklilu Lemma Institute of Pathobiology, Addis Ababa University. In addition, permission was obtained from the Research Committee of Bureau of Health, Amhara National Regional State, Ethiopia.

## 3. Results

### 3.1. Demographic and Clinical Characteristics of the Study Subjects

Data generated from 168 subjects were used in the analysis of the demographic and clinical results. Among the study participants, 52.4% were female, 73.8% were in age range of 18–39 years, 84.5% were new cases, 27.4% had a history of TB pertaining to one of their family members, and 67.9% were EPTB patients. Surprisingly, all EPTB cases were identified as TB lymphadenitis (TBLN), of which 67 (60.4%) and 18 (16.2%) were TBLN in cervical and axillary lymph nodes, respectively. Of the 168 isolates, 33.9% and 25.6% originated from South Gondar and West Gojjam, respectively (Table 1). Nonetheless, the sociodemographic and clinical characteristics of the patients did not affect the clustering rates and distribution of the lineages of MTBC strains (Table 1).

### 3.2. Spoligotyping Patterns of* Mycobacterium tuberculosis* Complex Strains

A total of 168 MTBC isolates were spoligotyped, and 89 (53%) different spoligotype patterns (strains) were identified. Clustering of isolates into strains was observed, and a total of 98 isolates were grouped in 19 (58.3%) different clusters of strains. The dominant strains were SIT289, SIT134, and SIT3411, each consisting of 28 (16.7%), 12 (7.14%), and 8 (4.76%) isolates, respectively. These strains contributed 28.6% (48/168) of all isolates with known spoligotype patterns. Out of the 89 spoligotype patterns (strains), 44 strains associated with 122 isolates matched the preexisting patterns in the SITVIT2 database while the remaining 45 spoligotype patterns associated with 46 isolates were not registered in the international spoligotype SITVIT2 database and thus designated as orphan strains.

Classification of MTBC strains showed the occurrence of the following lineages: Euro-American (86/168; 51.2%), East-African Indian (58/168; 34.5%),* M. africanum* (16/168; 9.52%), Indo-Oceanic (6/168; 3.57%), and* M. bovis* (2/168; 1.19%). Two of the spoligotypes (i.e., SIT982 and SIT665) were* M. bovis* and both were isolated from EPTB cases. Detailed spoligotyping results and their corresponding SITs/orphan strains and lineages are summarized in Tables 2 and 3.

### 3.3. Distribution of Strains and Lineages in the Study Area

**Figure 1:**
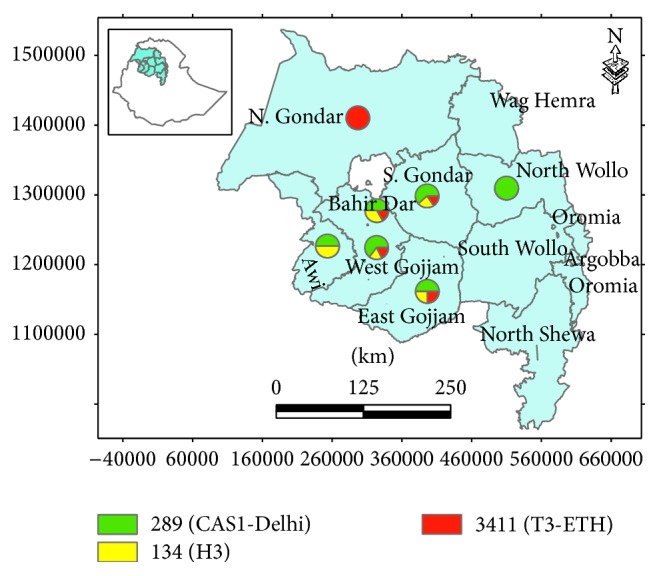
Map showing the distribution of the three dominant strains in Bahir Dar city and its surrounding zones, northwest Ethiopia. The green coloured portion of the icons in the map shows SIT289 with frequency of 14% (8/57) for South Gondar, 8.33% (2/24) for East Gojjam, 25.9% (11/43) for West Gojjam, 15% (3/20) for Bahir Dar Special Zone, and 15% (3/20) for Awi Zone. The yellow colour shows SIT134 with frequency of 5.26% (3/57) for South Gondar, 4.17% (1/24) for East Gojjam, 6.98% (3/43) for West Gojjam, 10% (2/20) for Bahir Dar Special Zone, and 15% (3/20) for Awi Zone. The red colour shows SIT3411 with frequency of 50% (1/2) for North Gondar, 3.51% (2/57) for South Gondar, 4.17% (1/24) for East Gojjam, 6.98% (3/43) for West Gojjam, and 5% (1/20) for Bahir Dar Special Zone.

The majority of MTBC strains were identified from the South Gondar Zone (57/168; 33.9%) followed by the West Gojjam Zone (43/168; 25.6%), each with a strain-clustering rate of 17.3% (Table 1). The distribution of the three dominant strains (SIT289, SIT134, and SIT3411) in the area is depicted in Figure 1.

**Figure 2:**
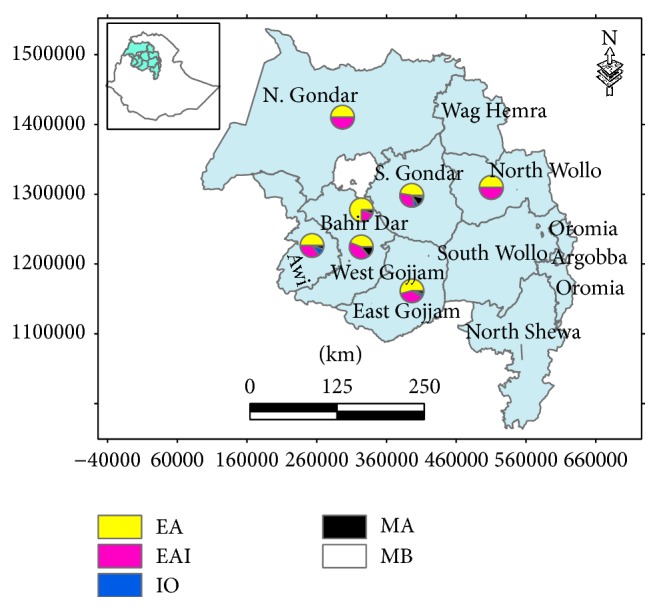
Distribution of mycobacterial lineages in the different zones of the Amhara Region, northwest Ethiopia. Five different lineages were identified. The Euro-American (EA) lineage is represented by yellow segments with a frequency of 50% (1/2) for North Gondar, 47.4% (27/57) for South Gondar, 54.2% (13/24) for East Gojjam, 44.2% (19/43) for West Gojjam, 75% (15/20) for Bahir Dar Special Zone, 50% (10/20) for Awi, and 50% (1/2) for North Wollo zone. The East-African Indian (EAI) lineage is represented by red segments with a frequency of 50% (1/2) for North Gondar, 31.6% (18/57) for South Gondar, 37.5% (9/24) for East Gojjam, 41.9% (18/43) for West Gojjam, 20% (4/20) for Bahir Dar Special Zone, 35% (2/20) for Awi, and 50% (1/2) for North Wollo zone. The Indo-Oceanic (IO) lineage is shown by blue segments with the frequency of 5.26% (3/57) for South Gondar, 4.17% (1/24) for East Gojjam, and 10% (2/20) for Awi Zone. The* M. africanum* (MA) lineage is represented by black segments with a frequency of 12.3% (7/57) for South Gondar, 4.17% (1/24) for East Gojjam, 14% (6/43) for West Gojjam, and 5% (1/20) each for Bahir Dar Special and Awi Zones.* M. bovis* (MB) was identified only in patients with TB lymphadenitis (TBLN) located in South Gondar with the prevalence of 3.51% (2/57) showed by a white coloured segment.

The Euro-American (EA), East-African-Indian (EAI), Indo-Oceanic (IO), and* M. africanum* (MA) lineages were identified in all study zones, whereas* M. bovis* (MB) was recorded only from patients with TBLN located in South Gondar.  Figure 2 depicts the distribution of the major lineages.

## 4. Discussion

In the present study, MTBC species were isolated from 168 TB patients from Bahir Dar city itself and the surrounding districts who visited health institutions in Bahir Dar city. The isolates were identified at strain and lineage levels on the basis of spoligotyping. Identification at a higher level of resolution by using variable nucleotide tandem repeat (VNTR) typing, facilitated by mycobacterial interspersed repetitive units- (MIRU-) VNTR, is desirable although this method is not currently available in Ethiopia.

Spoligotyping of 168 mycobacterial isolates revealed 89 distinct patterns, which corresponded to 53% of genotype diversity. The high diversity of spoligotypes strains that we observed in this study was consistent with the 59% reported by Tessema et al. [14] but higher than the percentages reported earlier by other studies in Ethiopia [3, 15–18]. This finding suggests the circulation of genetically variable strains in the study area, which could be the result of significant migration of infected people to Bahir Dar city and its surroundings from other regions of the country. In addition, the long period of MTBC clonal evolution may contribute to the diversity of strains [19]. Ninety-eight mycobacterial isolates were grouped into 19 clusters with an overall clustering percentage of 58.3%. The clustering rate observed in this study was slightly higher than those reported previously in Ethiopia [14, 18, 20]. On the other hand, it was lower than those reported by several other national studies [15, 16, 21] and international studies (e.g., South Africa [22] and Malawi [23]). The observed differences in clustering rates might be related to differences of sociocultural origin, sanitation, and population density. High level of strain clustering could suggest recent and ongoing TB transmission [24].

The prevalent strains identified in this study were SIT289, SIT134, and SIT3411. All three strains seem to be specific for the Bahir Dar city and its surroundings since they were not reported previously from other sites in Ethiopia [3, 14, 17, 18]. However, in the SITVIT database, SIT289 has only been reported from Brazil and Europe (mainly France, French Guiana, Martinique, Italy, Netherlands, and Sweden), while SIT134 has been reported from Central Asia and Middle East (Bangladesh, India, Pakistan, and Saudi Arabia), Australia, Netherlands, and United States of America [25]. A considerable number (46/168) of orphan strains were also recorded in this study. This is nearly identical to the average reported by Tessema et al. [14], Belay et al. [15], and Mihret et al. [16] in Ethiopia. The existence of mixed infections may also complicate spoligotyping results [6, 26], and hence higher resolution molecular tools should be applied toreveal thus far undefined mixed spoligotyping signatures.

Five different major lineages, namely, Euro-American, East-African-Indian, Indo-Oceanic,* M. africanum*, and* M. bovis*, were identified in this study. Euro-American was the dominant lineage, and more than half (51.5%) of the overall strains belonged to this lineage. This finding agreed with the results of previous studies in Ethiopia [3, 16–18] and Morocco [27]. The high proportion of new MTBC lineages is supposed to be related to their successful geographical spread as compared to ancient lineages [28, 29]. Even though Euro-American was identified as the prevalent major lineage, the CAS1_DELHI sublineage (consisting of 46 isolates) in the East-African Indian lineage appeared to have had a high transmission rate in our study population. This lineage is localized in South Asia, preferentially India, countries of the Middle East, and several other regions, including Africa [6]. It can be hypothesized that East-African Indian ancestral strains spread back from Asia to Africa through India as a result of human migration [30].

Screening of the SITVIT2 database also identified 9.52% (16/168) of the isolates as members of* M. africanum*. The clustering rate was 1.19% (2/168), indicative of a low rate of recent human-to-human transmission. Since it was not reported previously in Ethiopia [3, 14–18], isolation and identification of* M. africanum* in this study represent a novel finding. Further studies are needed to explore evolutionary aspects that may have contributed to the spread of* M. africanum* in the study population. Two strains of* M. bovis* (SIT982 and SIT665) were identified in this study. This finding was interesting and could implicate the public health importance of* M. bovis* in northwestern Ethiopia.

## 5. Conclusions

Molecular characterization of MTBC isolates from TB patients in Bahir Dar city and its surroundings was performed using spoligotyping. The high percentage of clustered strains of* M. tuberculosis* could suggest that a small number of lineages of* M. tuberculosis* are causing the disease in the area and isolation of* M. bovis* could suggest its zoonotic potential in the study area. Meanwhile, identification of* M. africanum* requires confirmation by molecular tools with a better discriminatory power than spoligotyping.

## Figures and Tables

**Table 1:** Demographic and clinical characteristics of the study subjects and their association with spoligotype clustering and major lineages (*n* = 168).

Characteristics	Number of isolates, *n* (%)	Number of isolates clustered versus unique ones				Major lineages by CBN					
		Clustered	Unique	Clustering rate	*P* value	EA	EAI	IO	MA	MB	*P* value
Patient origin (zonal)					0.502						0.822
North Gondar	2 (1.19)	1	1	0.6		1	1	0	0	0	
South Gondar	57 (33.9)	29	28	17.3		27	18	3	7	2	
East Gojjam	24 (14.3)	12	12	7.14		13	9	1	1	0	
West Gojjam	43 (25.6)	29	14	17.3		19	18	0	6	0	
Bahir Dar Special	20 (11.9)	13	7	7.74		15	4	0	1	0	
Awi	20 (11.9)	12	8	7.14		10	7	2	1	0	
North Wollo	2 (1.19)	2	0	1.19		1	1	0	0	0	
Age (years)					0.181						0.277
18–28	83 (49.4)	47	36	28		43	30	0	10	0	
29–39	41 (24.4)	28	13	16.7		19	16	2	3	1	
40–50	20 (11.9)	13	7	7.74		13	4	2	1	0	
>50	24 (14.3)	10	14	5.95		11	8	2	2	1	
Sex					0.175						0.674
Male	80 (47.6)	51	29	30.4		41	27	3	9	0	
Female	88 (52.4)	47	41	28		45	31	3	7	2	
Household size					0.855						0.876
≤4	85 (50.6)	49	36	29.2		42	31	2	9	1	
>4	83 (49.4)	49	34	29.2		44	27	4	7	1	
TB category					0.943						0.93
Retreatment	26 (15.5)	15	11	8.93		15	8	1	2	0	
New	142 (84.5)	83	59	49.4		71	50	5	14	2	
Family TB history					0.266						0.758
Yes	46 (27.4)	30	16	17.9		26	14	1	5	0	
No	122 (72.6)	68	54	40.5		60	44	5	11	2	
Clinical presentation					0.867						0.16
EPTB	114 (67.9)	67	47	39.9		53	43	6	10	2	
PTB	54 (32.1)	31	23	18.5		33	15	0	6	0	
Total	168 (100)	98	70	58.3		88	58	6	16	2	

EA: Euro-American; EAI: East-African Indian; IO: Indo-Oceanic; MA: *M. africanum*; MB: *M. bovis*; CBN: conformal Bayesian network; *P* values were presented at 95% confidence interval and *P* < 0.05 considered statistically significant.

**Table 2:** Spoligotype patterns of 44 shared types and their corresponding lineages/sublineages identified from a total of 168 *Mycobacterium tuberculosis* complex strains isolated in the Bahir Dar region.

SIT	Isolates with similar pattern	SITVIT2 lineage/sublineage	CBN^*∗*^ lineage	Octal number	Binary format
20	1	LAM1	EA	677777607760771	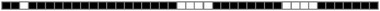
35	4	Ural-1	EA	777737777420771	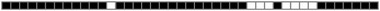
37	1	T3	EA	777737777760771	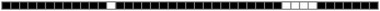
41	3	Turkey	EA	777777404760771	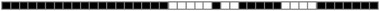
50	3	H3	EA	777777777720771	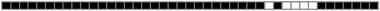
51	1	T	EA	777777777760700	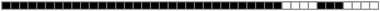
52	1	T2	EA	777777777760731	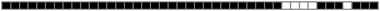
53	6	T	EA	777777777760771	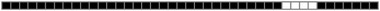
54	3	Manu2	EA	777777777763771	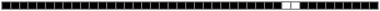
93	1	LAM5	EA	777737607760771	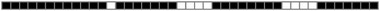
134	12	H3	EA	777777777720631	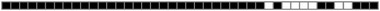
137	2	X2	EA	777776777760601	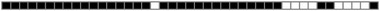
149	5	T3-ETH	EA	777000377760771	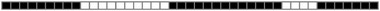
168	3	H3	EA	777777777720671	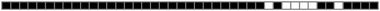
205	1	T	EA	737777777760771	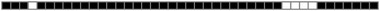
336	1	X1	EA	777776777760731	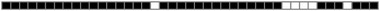
699	1	H3	EA	677777777720571	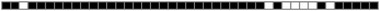
777	1	Ural-1	EA	777777777420771	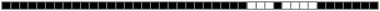
817	1	Ural-1	EA	777777777420731	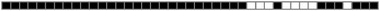
1166	1	T	EA	777377777760771	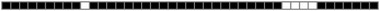
1552	1	H1	EA	777777774020631	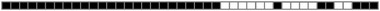
1688	1	T	EA	777777403760771	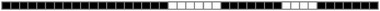
1877	1	T	EA	737377777760771	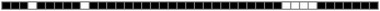
2007	1	T3	EA	777737677760771	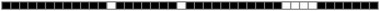
2409	1	T3	EA	777737757760771	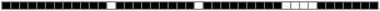
3134	1	H3	EA	777737377720771	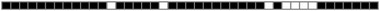
3411	8	T3-ETH	EA	777002377760771	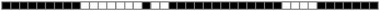
3412	2	T4	EA	777003377760771	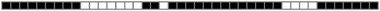
21	2	CAS1-Kili	EAI	703377400001771	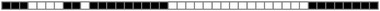
25	7	CAS1-Delhi	EAI	703777740003171	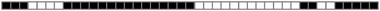
26	2	CAS1-Delhi	EAI	703777740003771	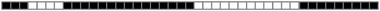
289	28	CAS1-Delhi	EAI	703777740003571	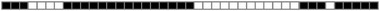
754	1	CAS1-Delhi	EAI	503777740003771	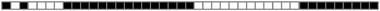
952	2	CAS1-Delhi	EAI	603777740003771	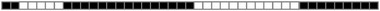
1200	2	Unknown	EAI	703777747777771	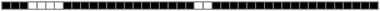
1551	1	CAS1-Delhi	EAI	701777740003771	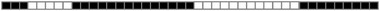
2359	2	CAS1-Delhi	EAI	703677740003171	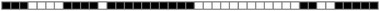
343	1	Unknown	*M. africanum*	700000007175771	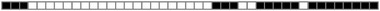
910	1	Unknown	*M. africanum*	700000007177771	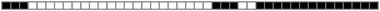
1729	1	Unknown	*M. africanum*	700000004177771	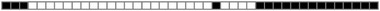
3409	1	AFRI	*M. africanum*	700020047177771	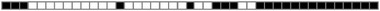
665	1	BOV_1	*M. bovis*	616773777777600	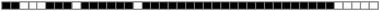
982	1	BOV	*M. bovis*	416773777777600	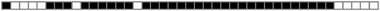
523	1	Manu_ancest	IO	777777777777771	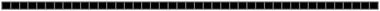

^*∗*^CBN: conformal Bayesian network; unknown: designates patterns with signatures that do not belong to any of the major lineages/sublineages described in the SITVIT2 database. The 168 isolates were grouped into 89 different spoligotype patterns (strains). Of the total 89 strains, 44 strains (patterns) have already been registered in the SITVIT2 database (Table 2), while the remaining 45 patters were orphans and presented in Table 3. The dominant strains were SIT289 (28 isolates), SIT134 (12 isolates), and SIT3411 (8 isolates) (Table 2). Furthermore, the 168 isolates were grouped into five different lineages including the Euro-American, East-African Indian, *M. africanum*, Indo-Oceanic, and *M. bovis* lineages in the order of decreasing percentage.

**Table 3:** Spoligotype patterns of 45 orphan strains and their corresponding lineages/sublineages identified from a total of 168 *Mycobacterium tuberculosis* complex isolates collected in tuberculosis patients in the Bahir Dar region.

SIT	Isolates with similar pattern	SITVIT2 lineage/sublineage	CBN^*∗*^ lineage	Octal number	Binary format
Orphan	1	T	EA	776603777760771	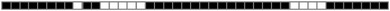
Orphan	1	T	EA	777737347760771	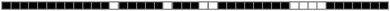
Orphan	1	T	EA	276777777760771	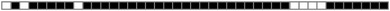
Orphan	1	T3-ETH	EA	777002377420771	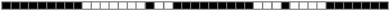
Orphan	1	T2	EA	777777403760731	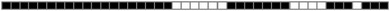
Orphan	1	EAI	EA	777760370000000	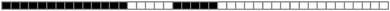
Orphan	1	EAI	EA	777770370000000	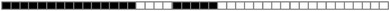
Orphan	1	T3-ETH	EA	777002377760731	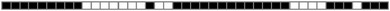
Orphan	1	T1-RUS2	EA	770002001760771	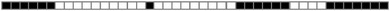
Orphan	1	EAI	EA	777760370000000	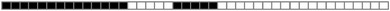
Orphan	1	T	EA	276777737760771	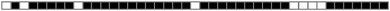
Orphan	1	T-H37Rv	EA	777777444760771	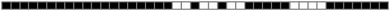
Orphan	1	X1	EA	400002757760771	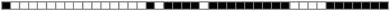
Orphan	1	T2	EA	777777403760731	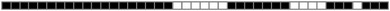
Orphan	1	Manu2	EA	577747777767771	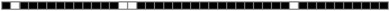
Orphan	1	H3	EA	777737377720731	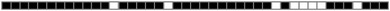
Orphan	1	Manu2	EA	777777774363771	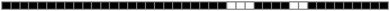
Orphan	1	LAM3	EA	760002007760771	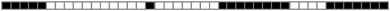
Orphan	1	AFRI	EAI	700022044037771	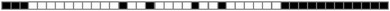
Orphan	1	Unknown	EAI	000022000003771	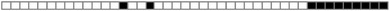
Orphan	1	CAS	EAI	700002000000771	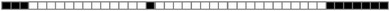
Orphan	1	PINI2	EAI	400200000000751	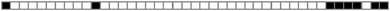
Orphan	1	H2	EAI	700000004037771	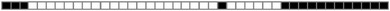
Orphan	1	CAS1-Delhi	EAI	703602040003571	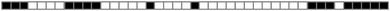
Orphan	1	PINI2	EAI	000022000003771	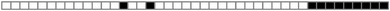
Orphan	1	H	EAI	000002004020631	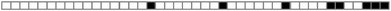
Orphan	1	CAS1-Delhi	EAI	703677740003571	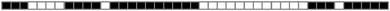
Orphan	1	CAS	EAI	703777700001171	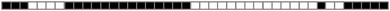
Orphan	1	CAS1-Delhi	EAI	703622040003571	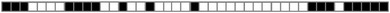
Orphan	1	AFRI	*M. africanum*	700022007177771	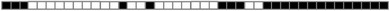
Orphan	1	AFRI	*M. africanum*	700002044177771	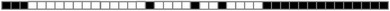
Orphan	1	AFRI	*M. africanum*	700020044177771	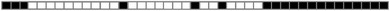
Orphan	2	AFRI	*M. africanum*	700002004177771	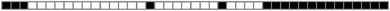
Orphan	1	AFRI	*M. africanum*	700002004177771	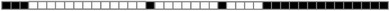
Orphan	1	AFRI	*M. africanum*	771022044177771	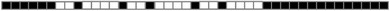
Orphan	1	AFRI	*M. africanum*	700002004177771	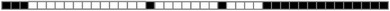
Orphan	1	AFRI	*M. africanum*	700002007177771	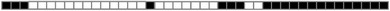
Orphan	1	AFRI	*M. africanum*	700002007177771	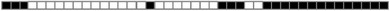
Orphan	1	AFRI	*M. africanum*	700022004177771	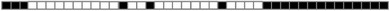
Orphan	1	AFRI	*M. africanum*	700022047177771	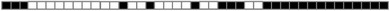
Orphan	1	Manu1	IO	773777747777771	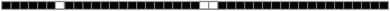
Orphan	1	Manu2	IO	773777744203771	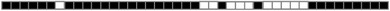
Orphan	1	Manu1	IO	753777747777771	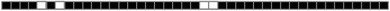
Orphan	1	Manu1	IO	717777777777771	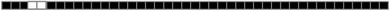
Orphan	1	EAI	IO	717777776003771	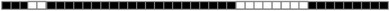

^*∗*^CBN: conformal Bayesian network; unknown: designates patterns with signatures that do not belong to any of the major lineages/sublineages described in the SITVIT2 database. Forty-five of the total 89 strains were identified as orphan strains in the present study and shown in Table 3. The orphan strains belonged to four lineages including Euro-American, East-African Indian, *M. africanum*, and Indo-Oceanic lineages.
